# CRISPRi as a Tool to Repress Multiple Copies of Extracellular Polymeric Substances (EPS)-Related Genes in the Cyanobacterium *Synechocystis* sp. PCC 6803

**DOI:** 10.3390/life11111198

**Published:** 2021-11-06

**Authors:** Marina Santos, Catarina C. Pacheco, Lun Yao, Elton P. Hudson, Paula Tamagnini

**Affiliations:** 1 i3S-Instituto de Investigação e Inovação em Saúde, Universidade do Porto, 4000-008 Porto, Portugal; marina.santos@ibmc.up.pt (M.S.); cclopes@ibmc.up.pt (C.C.P.); 2 IBMC-Instituto de Biologia Molecular e Celular, Universidade do Porto, 4000-008 Porto, Portugal; 3 Programa Doutoral em Biologia Molecular e Celular (MCbiology), Instituto de Ciências Biomédicas Abel Salazar (ICBAS), Universidade do Porto, 4000-008 Porto, Portugal; 4 Science for Life Laboratory, KTH Royal Institute of Technology, 10004 Stockholm, Sweden; lunyao@kth.se (L.Y.); paul.hudson@scilifelab.se (E.P.H.); 5 Department of Protein Science, KTH Royal Institute of Technology, 10004 Stockholm, Sweden; 6 Departamento de Biologia, Faculdade de Ciências, Universidade do Porto, 4000-008 Porto, Portugal

**Keywords:** CRISPRi, cyanobacteria, *Synechocystis*, extracellular polymeric substances (EPS), KpsM

## Abstract

The use of the versatile cyanobacterial extracellular polymeric substances (EPS) for biotechnological/biomedical applications implies an extensive knowledge of their biosynthetic pathways to improve/control polymer production yields and characteristics. The multiple copies of EPS-related genes, scattered throughout cyanobacterial genomes, adds another layer of complexity, making these studies challenging and time-consuming. Usually, this issue would be tackled by generating deletion mutants, a process that in cyanobacteria is also hindered by the polyploidy. Thus, the use of the CRISPRi multiplex system constitutes an efficient approach to addressing this redundancy. Here, three putative *Synechocystis* sp. PCC 6803 *kpsM* homologues (*slr0977*, *slr2107*, and *sll0574*) were repressed using this methodology. The characterization of the 3-sgRNA mutant in terms of fitness/growth and total carbohydrates, released and capsular polysaccharides, and its comparison with previously generated single knockout mutants pointed towards Slr0977 being the key KpsM player in *Synechocystis* EPS production. This work validates CRISPRi as a powerful tool to unravel cyanobacterial complex EPS biosynthetic pathways expediting this type of studies.

## 1. Introduction

Most of the cyanobacterial strains can produce extracellular polymeric substances (EPS), mainly composed of heteropolysaccharides, that can be released to the extracellular medium or remain associated to the cell surface as capsules, sheaths, or slime. [1,2]. These EPS are emerging as promising biomaterials for biotechnological and biomedical applications as they possess distinctive and advantageous characteristics compared to other natural and synthetic polymers [3,4,5]. In addition to the complexity/variety of polymers produced by cyanobacteria, their cultivation is inexpensive due to their photoautotrophic lifestyle, the growth rates are similar or higher than algae and plants, and often, the produced polymers can be easily functionalized and the producing strain engineered to obtain a product with the desired properties and/or enhanced performance [6]. However, this manipulation requires a comprehensive knowledge on the molecular mechanisms underlying EPS biosynthesis, assembly, and export. Such knowledge is therefore crucial to increase the production yields and to tailor polymer variants for a specific application. In addition, this knowledge can also aid efforts to redirect carbon fluxes from the high-energy-demanding EPS production towards the production of target/value-added compounds, when using cyanobacteria as green cell factories [7,8].

The last steps of the EPS biosynthetic pathways are relatively conserved throughout bacteria and often follow one of three major pathways—Wzy-, ABC transporter- or Synthase-dependent—ending with an assembled polymer outside the cell wall [9]. Previously, by performing a phylum-wide analysis, we showed that most cyanobacteria harbor genes encoding proteins related to these pathways but often not a complete set defining one pathway, and the EPS-related genes are scattered throughout the genomes or organized in small clusters, often in multiple copies [1,10,11]. Up until now, the study of the putative EPS-related genes/proteins has been performed, by us and others, mainly through the generation and characterization of knockout mutants using the model cyanobacterium *Synechocystis* sp. PCC 6803 (hereafter *Synechocystis*). Previous works have confirmed the involvement of cyanobacterial homologues of key bacterial proteins belonging to both the ABC transporter- and Wzy-dependent pathways in EPS production. Regarding the Wzy-dependent pathway, knockout mutants of *wza* (*sll1581*), *wzb* (*slr0328*), and *wzc* (*sll0923*) exhibited fewer capsular polysaccharides (CPS), fewer released polysaccharides (RPS), or less of both, respectively [12,13]. Regarding the ABC transporter-dependent pathway, Fisher et al. reported knockout mutants of a putative transport permease (*slr0977* (*kpsM*)) and its associated ATP-binding module (*slr0982* (*kpsT*)), and a triple mutant (*slr0977* (*kpsM*) and the putative pair *sll0574* (*kpsM*)/*sll0575* (*kpsT*)) produced EPS with different monosaccharidic composition compared to the EPS produced by the wild-type [14]. While Fisher et al. [14] did not report on the amounts of EPS produced by these mutants, a recent extensive characterization of a *slr0977* (*kpsM*) mutant showed that the absence of Slr0977 resulted in a significant reduction of RPS (50%) and a smaller decrease of CPS (20%) [8]. In addition, a mutant lacking Slr2107 (another KpsM homologue) did not show significant differences in the total carbohydrates, RPS, and CPS compared to the wild-type [13]. Although one must bear in mind the growth conditions and the *Synechocystis* substrain used in those studies, disruption of *slr0977* (*kpsM*) is thus responsible for one of the most significant reductions in the amount of RPS reported to date.

**Figure 1:**
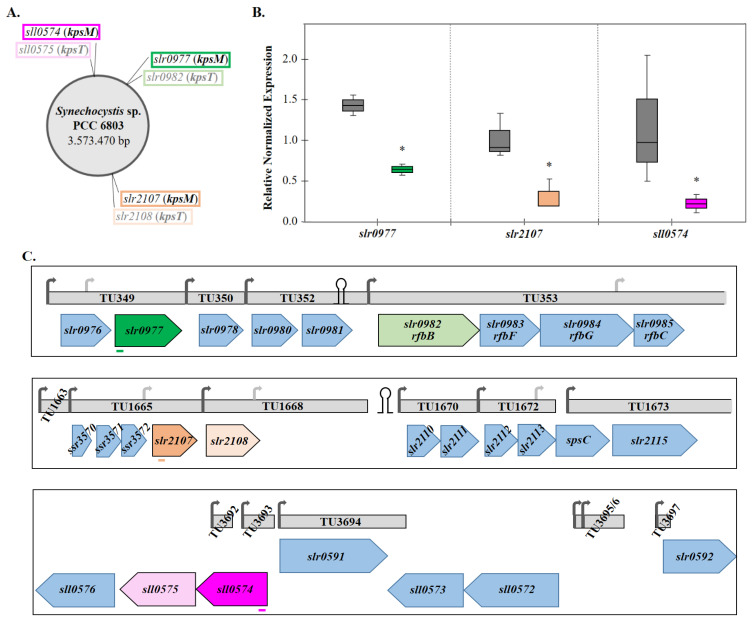
EPS-related genes in *Synechocystis* sp. PCC 6803 encoding a putative transport permease of the ABC transporter-dependent pathway. (**A**) Location of the putative homologues of *kpsM*/*kpsT* in *Synechocystis* chromosome (*kpsT* is the second component of the two-protein complex, and is responsible for ATP-binding). (**B**) CRISPRi multiplex repression of three *kpsM* homologues (*sll0574*, *slr0977*, and *slr2107*), evaluated by RT-qPCR. The catalytically dead Cas9 (dCas9) and the 3 single guide-RNAs were constitutively expressed from promoters PpsbA2 and PL31, respectively. Expression of the target genes in the 3-sgRNA mutant (*slr0977*: green; *slr2107*: orange; *sll0574*: pink) relative to *Synechocystis* wild-type harboring dCas9 (grey). Data from at least two biological replicates and three technical replicates were normalized against three reference genes (*rrn16S*, *rnpB*, and *petB*), the whiskers represent the minimum and maximum non-outlier values in the data set. * *p*-value ≤ 0.05. (**C**) Schematic representation of the genomic context of the three target genes. sgRNA binding sites are depicted as colored lines below the target gene. Neighboring genes are annotated according to information available at the CyanoBase and KEGG databases. The transcriptional unit and transcription start sites (arrows; light grey indicate internal TSSs) are annotated according to Kopf et al. [25]. The predicted terminators (loops) were found using the FindTerm algorithm (Softberry).

Nevertheless, it is important to highlight that *kpsM* has three putative homologues in *Synechocystis*: *slr0977*, *slr2107*, and *sll0574* (Figure 1A), and it is necessary to clarify the role of the proteins, encoded by these genes, on EPS production. Traditionally, this would be tackled by generating a triple knockout mutant. However, the systematic knockout of multiple genes in *Synechocystis* is a time-consuming task due to its relatively slow growth rate compared to other bacteria, polyploidy [15], and the need to use different and increasing concentrations of antibiotics as selection markers. The use of a system such as CRISPR/Cas (clustered regularly interspaced short palindromic repeats/CRISPR associated nuclease) allows faster and easier gene editing, cleavage, or inactivation [16]. In addition, CRISPRi (interference), which relies on the use of a nuclease-deficient Cas9 (dCas9) and a single-guide RNA (sgRNA), enables targeted gene regulation as the dCas9-sgRNA complex blocks the RNA polymerase binding or the elongation, resulting in gene repression. The main advantage of CRISPRi over traditional gene knockouts is the ability to repress multiple genes simultaneously, as elegantly demonstrated in 2016 by Yao et al. [17] that reported the repression of up to four genes, providing the CRISPRi multiplex proof-of-concept for cyanobacteria.

In this work, to pursue the unravelling of cyanobacterial EPS assembly and export pathways, the CRISPRi system was employed as a tool for the multiplex repression of EPS-related genes in *Synechocystis*, namely for the three *kpsM* homologues (*slr0977*, *slr2107* and *sll0574*). The generated mutant was characterized in terms of growth and carbohydrate production, and its phenotype compared to the conventional single knockout mutants generated by double homologous recombination.

## 2. Experimental Section

To construct the strain that will serve as a control and genetic background for the CRISPRi experiments (*Syn* dCas9), *Synechocystis* sp. PCC 6803 (Pasteur Culture Collection), substrain Kazusa [18,19] was transformed with the pMD19T vector harboring the sequence encoding the dCas9 from *Streptococcus pyogenes* under the control of the constitutive promoter PpsbA2. This construct was integrated into the *psbA1* neutral site of the *Synechocystis* chromosome (Figure S1). For the simultaneous repression of the three putative *kpsM* homologues (*slr0977*, *slr2107*, and *sll0574*), an array of 3-sgRNAs was designed based on Larson et al. [20] and constructed as described [17]. Each 100 bp sgRNA unit comprised (i) its own promoter, (ii) the dCas9 binding handle and protospacer, and (iii) a terminator. The designed sgRNAs (Table 1) were evaluated for potential off-target binding sites using the CasOT software [21]. All the potential off-targets detected contained 6 or more mismatches compared to the sgRNA protospacer, including at least 1 in the 12 bp seed region (Table S1), so off-target binding was likely not significant. Therefore, these sgRNAs were expressed constitutively using the PL31 promoter (without a TetR repressor) and introduced into *Syn* dCas9 using a pLYK2-derived replicative plasmid (Table S2).

The multiplex repression of the *kpsM* homologues was quantified by RT-qPCR, assessing the expression of *slr0977*, *slr2107*, and *sll0574* in the 3-sgRNA *kpsM* mutant compared to the *Syn* dCas9. Sample collection (using three clones of the 3-sgRNA *kpsM* mutant), RNA extraction, cDNA synthesis, as well as the control measurements and PCRs were performed as previously described [22], except that three-fold standard dilutions of the cDNAs were made (1/3, 1/9, 1/27 and 1/81). The RT-qPCR reactions (10 µL) were setup as described by Pinto et al. [23] using the iTaq™ Universal SYBR^®^ Green Supermix (Bio-Rad(Hercules, CA, USA)), 1 μL of template cDNA and the primers listed in Table S3. Validation of the reference genes (*rrn16S*, *petB*, and *rnpB*) and data analysis were performed using the Bio-Rad CFX Maestro™ 1.1 software. The single *sll0574* knockout mutant was generated via double homologous recombination, by partially replacing the gene with a kanamycin (Km) resistance cassette, as described by Santos et al. [8]. The mutants were characterized in terms of growth (absorbance and chlorophyll *a* content) and carbohydrate production (total carbohydrates, CPS, and RPS) using the phenol-sulfuric acid method [24], as described previously by Santos et al. [8]. Data were statistically analyzed with GraphPad Prism v5 (GraphPad Software) using analysis of variance (ANOVA), followed by Tukey’s multiple-comparison test. For the qPCR data, analysis was performed using CFX Maestro Software (Bio-Rad) using analysis of variance (ANOVA).

## 3. Results & Discussion

### Repression of Three kpsM Homologues in Synechocystis and Characterization of the 3-sgRNA Mutant

The *kpsM* homologues (*slr0977*, *slr2107*, and *sll0574*) were successfully repressed in the *Synechocystis* 3-sgRNA *kpsM* mutant compared to *Syn* dCas9 (control strain). The repression levels for the target genes were: ~60% for *slr0977*, ~70% for *slr2107*, and ~80% for *sll0574* (Figure 1B). Since the repression of *slr0977* was weaker compared to the other targets, the integrity of the sgRNAs in all clones was confirmed by sequencing. The repression level observed could be explained by *slr0977* being the second gene in its operon (Figure 1C). The *slr0977* predicted transcription start site (TSS) is 1175 bp upstream from the start codon [25] and 1191 bp upstream from the sgRNA binding site (Figure 1C). As mentioned by Yao et al. [17], the blocking of transcription elongation may not be as efficient if the gene of interest is part of an operon with a distant TSS. Nevertheless, the repression levels obtained in this study are within those previously reported for other targets in *Synechocystis* [17,26,27]. On the one hand, it is also possible to purposefully design the sgRNA further from the TSS, resulting in lower repression of the target gene(s) and enabling fine-tuning gene expression, as demonstrated by Shabestary et al. [26]. On the other hand, a partial repression will allow to sustain cell viability, even when essential genes are targeted, allowing the identification of new phenotypes.

**Figure 2:**
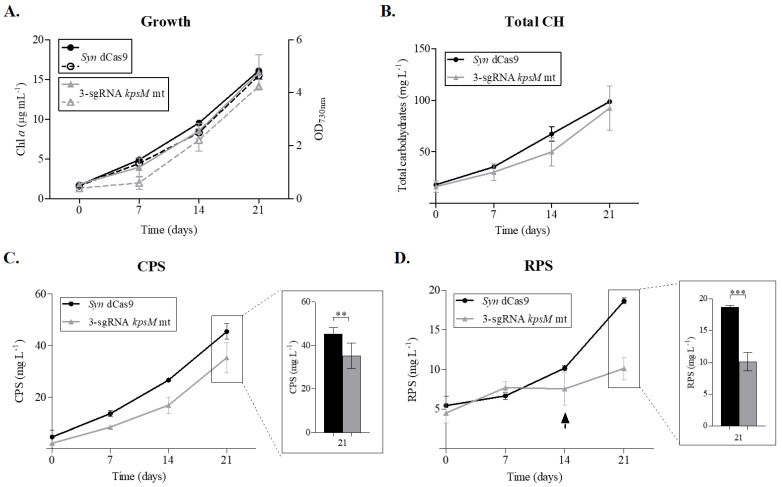
Growth curves and carbohydrate production by *Synechocystis* sp. PCC 6803 constitutively expressing the dead Cas 9 (*Syn* dCas9), and the 3 single guide-RNAs *kpsM* mutant targeting *sll0574*, *slr0977* and *slr2107* (3-sgRNA *kpsM* mt). Growth was monitored by measuring the optical density at 730 nm (full lines) and chlorophyll *a* (Chl *a*) (dashed lines) (**A**). Total carbohydrates (Total CH) (**B**), capsular polysaccharides (CPS) (**C**), and released polysaccharides (RPS) (**D**) were measured by the phenol-sulfuric acid method [24] and expressed as milligrams per liter of culture. The arrow indicates the point of divergence between the amount of RPS of *Syn* dCas9 and the 3-sgRNA *kpsM* mutant. Cells were grown in BG11 medium at 30 °C under a 12 h light (50 µE m^−2^ s^−1^)/12 h dark regimen, with orbital shaking at 150 rpm. Experiments were performed in triplicate, and statistical analysis is presented for the final time point (** *p-value* ≤ 0.01 *** *p*-value < 0.001).

Subsequently, the 3-sgRNA *kpsM* mutant was characterized in terms of growth and carbohydrate production. Repression of the *kpsM* homologues did not significantly affect growth, compared to the *Syn* dCas9 (Figure 2A) and wild-type strains. However, similarly to the *slr0977* knockout mutant, the 3-sgRNA *kpsM* mutant displayed a clumping phenotype at low cell densities [8]. Regarding total carbohydrates, the 3-sgRNA *kpsM* mutant produced approximately the same amount as the *Syn* dCas9 strain (Figure 2B). However, it had approximately 20% less CPS and 40% less RPS at 21 days of cultivation (Figure 2C,D).

This phenotype is very similar to the one observed for the *slr0977* single knockout mutant [8], suggesting that the protein encoded by *slr0977* could be the main KpsM homologue involved in RPS export, at least in the conditions tested. However, in the *slr0977* single mutant, at 21 days, the amount of RPS is 50% less compared to the wild-type [8], while in the 3-sgRNA *kpsM* mutant, this difference only reaches 40%. In addition, while the amount of RPS for the *slr0977* mutant is already reduced at the start of the experiment [8], in the 3-sgRNA *kpsM* mutant, this asymmetry is only noticeable after 14 days of cultivation (Figure 2D, arrow), which could be due to the weaker level of repression achieved for *slr0977* (60%). This reduction on RPS production occurs without a significant change in the amount of total carbohydrates. In the single *slr0977* mutant, this is associated with the intracellular accumulation of poly-hydroxybutyrate (PHB) [8], as it may happen in the 3-sgRNA *kpsM* mutant.

**Figure 3:**
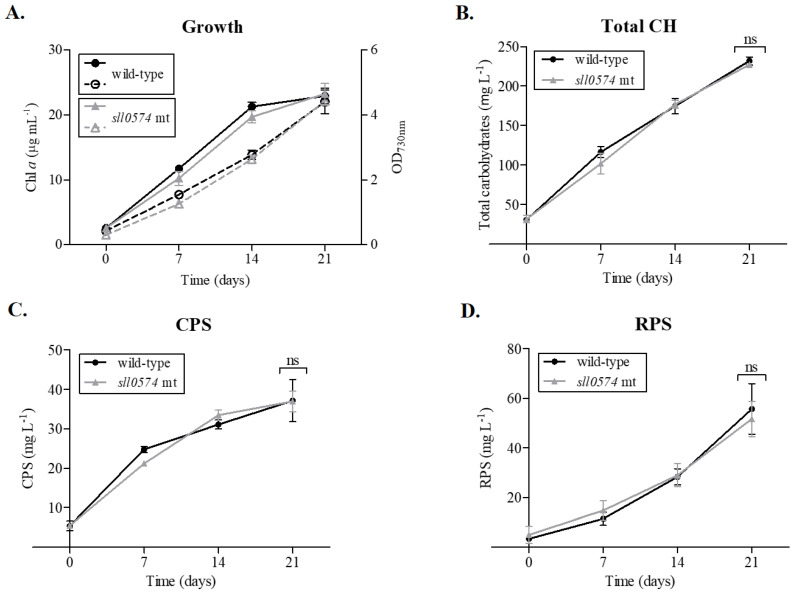
Growth curves and carbohydrate production by *Synechocystis* sp. PCC 6803 wild-type and the *kpsM sll0574* mutant (*sll0574* mt). Growth was monitored by measuring the optical density at 730 nm (full lines) and chlorophyll *a* (Chl *a*) (dashed lines) (**A**). Total carbohydrates (Total CH) (**B**), capsular polysaccharides (CPS) (**C**), and released polysaccharides (RPS) (**D**) were measured by the phenol-sulphuric acid method [24] and expressed as milligrams per liter of culture. Cells were grown in BG11 medium at 30 °C under a 12 h light (50 µE m^−2^ s^−1^)/12 h dark regimen, with orbital shaking at 150 rpm. Experiments were performed in triplicate, and statistical analysis is presented for the final time point (ns: not significant; *p*-value > 0.05).

To our knowledge, no single mutant on the third *kpsM* homologue, *sll0574*, had been previously generated. Therefore, we generated a *sll0574* knockout mutant by partially replacing the gene with a kanamycin (Km) resistance cassette via double homologous recombination (Table S2) and characterized it in terms of its growth and carbohydrate content. The *sll0574* mutant did not show any significant differences in growth, total carbohydrates, RPS, or CPS compared to the wild-type (Figure 3), as it was previously reported for the *slr2107* mutant [13]. CRISPRi mutants for each target gene were not generated, as the goal was to evaluate the effect of the simultaneous repression of the three *kpsM* homologues. Although each sgRNA could have off-target effects (detailed analysis in Table S1), Yao et al. have previously shown that the expression of dCas9 from various weak and moderate promoters with a non-targeting, “dummy” sgRNA does not significantly affect the growth of *Synechocystis* or its transcriptome, suggesting that off-target binding with phenotypical consequences is indeed infrequent [17,28]. A direct comparison between deletion and repression mutants is not straightforward; however, the phenotype shared by the 3-sgRNA *kpsM* and *slr0977* mutants, together with the absence of an EPS-related phenotype for the *slr2107* and *sll0574* single knockout mutants, further supports our hypothesis that Slr0977 is the key KpsM homologue involved in RPS export, at least under the conditions tested. In agreement, a comparative analysis of the transcriptomes of *Synechocystis* under ten different conditions [25] showed that *slr0977* was indeed the most expressed, while the *slr2107* transcript levels increased under specific stress conditions (low temperature and nitrogen depletion). In Kopf et al., no data were reported for *sll0574* (consistent with the low levels detected in our RT-qPCR experiment).

## 4. Conclusions

In summary, the use of CRISPRi in multiplex to repress the three putative *kpsM* homologues in *Synechocystis* established a novel approach to tackle the redundancy of EPS-related genes. The use of this methodology not only expands the possibilities to study other putative redundant components, but also the simultaneous evaluation of homologues that in other bacteria are associated with the different pathways. As we expect that the cyanobacterial EPS biosynthetic pathways will diverge from the well-characterized bacterial ones, the use of CRISPRi will enable a faster screening of the role of the different players, allowing to piece together these molecular mechanisms. Although the use of CRISPRi in cyanobacteria is not yet widespread, this platform is certainly a powerful tool and will become more relevant as it is more frequently used.

## Figures and Tables

**Table 1:** Sequences of the three sgRNAs used in this study.

sgRNA Identifier/(Position) *	sgRNA Sequence Including PAM #
sll0574 (15)	GGGGACCAGTTCACCCTTGTCGG
slr0977 (16)	CCCCCAGAACTGATTATTGAAGCAGGAC
slr2107 (56)	CCCATGACTGGTTGCGATTGACGAT

* sgRNA nucleotide binding position counting from the start codon. # underlined nucleotides indicate the protospacer adjacent motif (PAM).

## Data Availability

Not applicable.

## Supplementary Materials

The following are available online at https://www.mdpi.com/article/10.3390/life11111198/s1: Figure S1: *Synechocystis* sp. PCC 6803 wild-type *slr1181* (*psbA1*) genomic context and confirmation of the *Syn* dCas9 mutant generation. Table S1: list of potential off-target binding sites for the three sgRNAs used in this work; Table S2: list of organisms and plasmids used/generated in this work; Table S3: primer nucleotide sequences and annealing temperatures (Ta) used in RT-qPCR; Table S4: Primer nucleotide sequences used to verify the segregation of the *Syn* dCas9 strain.
