# Supplementary material for: CRISPRi as a Tool to Repress Multiple Copies of Extracellular Polymeric Substances (EPS)-Related Genes in the Cyanobacterium Synechocystis sp. PCC 6803
Source: Life (Basel). 2021 Nov 6;11(11):1198. doi: 10.3390/life11111198 (PMC8620461; doi:10.3390/life11111198)
Supplement: Supplementary file 1 [file life-11-01198-s001.zip › life-1446754-supplementary.pdf]

life

Supplementary Materials: CRISPRi as a Tool to Repress Multiple Copies of Extracellular Polymeric Substances (EPS)-Related Genes in the Cyanobacterium *Synechocystis* sp. PCC 6803

Marina Santos [1,2,3], Catarina C. Pacheco [1,2], Lun Yao [4,5,†], Elton P. Hudson [4,5] and Paula Tamagnini [1,2,6,*]

1. i3S- Instituto de Investigação e Inovação em Saúde, Universidade do Porto, 4000-008 Porto, Portugal; marina.santos@ibmc.up.pt (M.S.); cclopes@ibmc.up.pt (C.C.P)
2. IBMC- Instituto de Biologia Molecular e Celular, Universidade do Porto, 4000-008 Porto, Portugal
3. Programa Doutoral em Biologia Molecular e Celular (MCbiology), Instituto de Ciências Biomédicas Abel Salazar (ICBAS), Universidade do Porto, 4000-008 Porto, Portugal
4. Science for Life Laboratory, KTH Royal Institute of Technology, 10004 Stockholm, Sweden; lunyao@kth.se (L.Y.); paul.hudson@scilifelab.se (E.P.H)
5. Department of Protein Science, KTH Royal Institute of Technology, 10004 Stockholm, Sweden
6. Departamento de Biologia, Faculdade de Ciências, Universidade do Porto, 4000-008 Porto, Portugal

* Correspondence: pmtamagn@ibmc.up.pt, Tel.: +351 226074957

† Present Address: Dalian Institute of Chemical Physics, Dalian 116000, Shandong, China

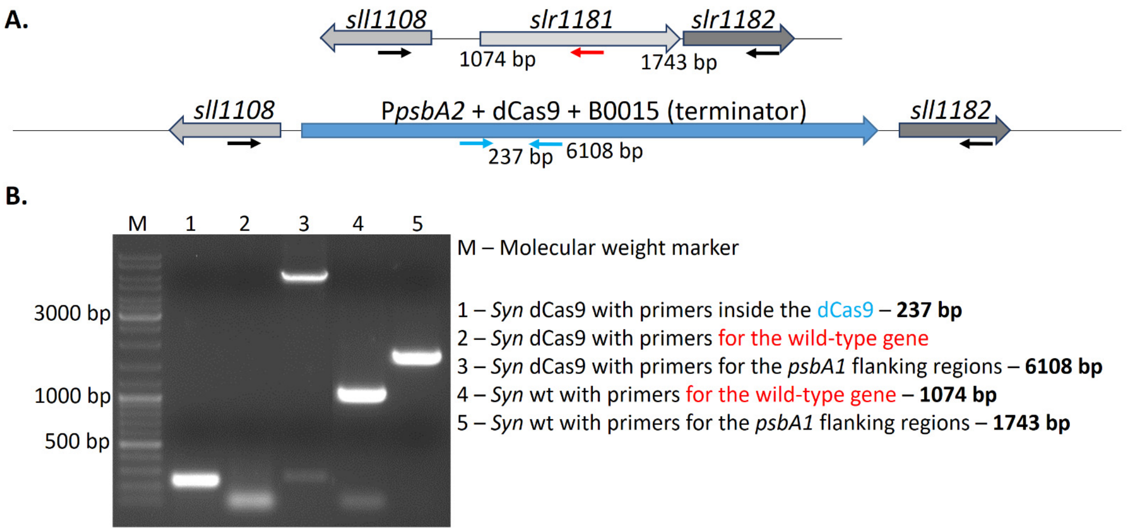

Figure S1. *Synechocystis* sp. PCC 6803 wild-type *slr1181* (*psbA1*) genomic context and confirmation of the *Syn* dCas9 mutant generation. (**A**) Schematic representation of the *slr1181* locus in the genome of *Synechocystis*. Arrowheads—oligonucleotides used to assess mutant identity and segregation (Table S4). (**B**) GreenSafe stained agarose gel showing PCR analyses to assess chromosome segregation of *Syn* dCas9 mutant. PCRs were carried out by using genomic DNA extracted from *Synechocystis* wild-type (*Syn* wt) (Lanes 4 and 5) or the *Syn* dCas9 mutant (Lanes 1,2 and 3) as template. M, GeneRuler DNA ladder mix (Thermo Scientific). The sizes in base-pairs (bp) of some of the GeneRuler DNA Ladder Mix fragments are shown for reference on the left.

Table S1. List of potential off-target binding sites for the three sgRNAs used in this work.

Target	Chromosome Location	Strand	Site	Mismatch (Mm) Type	All Mm	Target	Feature
slr0977	180238-180266	-	tTaaccagatcta_AATCAGcgCTGG	A212	13	*slr1414*	sensory transduction histidine kinase
	283005-283033	-	cggagcagatcAT_AATCctTTCTGG	A211	13	-	-
	307315-307343	+	GaCgaatgcCcAg_AAcCAGTgCTGG	A209	11	*slr1303*	unknown protein
	528989-529017	+	agaCacCaTtAcc_AATCAacTCTGG	A209	11	*slr1753*	unknown protein
	604729-604757	+	cTtaTctTcCtgT_AAaCAGTgCTGG	A208	10	-	-

	609572-609600	-	aTagctCcaCAtc_AAcCAGTTtTGG	A209	11	*slr1906*	unknown protein
	709902-709930	-	GaatctggTgAca_AAgaAGTTCTGG	A210	12	-	-
	714970-714998	-	caagaatcgCcca_AATCAGTTCaaG	A212	14	*sll1583*	DNA ligase
	804862-804890	+	tTttccCaTggca_AATCAtTTCTGc	A210	12	*sll1076*	Zinc exporter
	816533-816561	+	tggagGtcgtAgg_AAgCAGTgCTGG	A211	13	*sll1072*	unknown protein
	1479022-1479050	-	taataatcgttta_AAcCAtTTCTGG	A213	15	*slr1724*	unknown protein
	1643383-1643411	-	aattTGgcaCctT_AAcCAGTTCTtG	A209	11	-	-
	1724715-1724743	+	GcgtaGaTaCggc_AATCAGcaCTGG	A209	11	*sll1258*	unknown protein
	1887090-1887118	-	agCaatCagCtca_AAcCcGTTCTGG	A210	12	-	-
	2076362-2076390	+	taaCcatccaAcc_AgTCAGTgCTGG	A211	13	*sll1522*	CDP-diacylglycerol-glycerol-3-phosphate 3phosphatidyltransferase
	2228027-2228055	+	catCccCagggAa_AATtAGTTCgGG	A210	12	*slr1933*	dTDP-4-dehydrorhamnose 3,5-epimerase
	2438430-2438458	-	ccCagGggcaAtg_AATtAGTTtTGG	A210	12	*sll0319*	unknown protein
	2735371-2735399	+	ccCCatggcCgca_AcTCAGTTaTGG	A210	12	-	-
	2782435-2782463	-	tTttctaaTaAgT_AATCAaTTtTGG	A209	11	*slr0907*	unknown protein
	3142190-3142218	+	taaaactTTtcta_AATCAGTgCcGG	A211	13	*sll0045*	sucrose phosphate synthase
	3231946-3231974	-	tTaCccacTgcAa_AATCtGTTCcGG	A209	11	*slr0930*	unknown protein
slr2107	46664-46689	-	tgctCTatga_cCGATgGACGAT	A208	10	*slr1494*	ABC transporter
	312905-312930	-	AgaggattTg_GCGATcGcCGAT	A208	10	*slr1306*	unknown protein
	355492-355517	-	AaaAtccccg_GCGATcGcCGAT	A208	10	*slr0985*	dTDP-6-deoxy-L-mannose-dehydrogenase
	482785-482810	-	tTacCTtccT_GCcATTGACGgT	A206	8	-	-
	538261-538286	-	gacAtgGtcg_GCGATcGcCGAT	A208	10	*slr1760*	regulatory components of sensory transduction system
	674519-674544	-	gcaAaaaaTT_GCGgTTGACGAc	A207	9	*slr1379*	cytochrome oxidase d subunit I
	932640-932665	+	ATtAtTGcgg_GCctTTGACGAT	A205	7	*slr1829*	polyhydroxyalkanoate synthase subunit PhaE
	1458346-1458371	-	ccatCaccaT_GCGATTGACcAc	A208	10	*sll1614*	cation-transporting ATPase
	1586617-1586642	+	Aaattcaccg_GCaATTGACGAT	A109	10	*sll1425*	proline-tRNA ligase
	1763748-1763773	-	AgttCcaGcg_GCGATcGcCGAT	A207	9	*slr1962*	unknown protein
	1935017-1935042	-	cattgccaaa_GCcATTGACGcT	A210	12	*sll1091*	bacteriochlorophyll synthase subunit
	1944159-1944184	-	cgtttTtGca_cCGtTTGACGAT	A208	10	*slr1173*	unknown protein
	1960433-1960458	+	ATacCcaGTT_GgGgTTGACGAT	A204	6	*sll1564*	alpha-isopropylmalate synthase
	2154990-2155015	-	cccAtaacaa_GCGATcGgCGAT	A209	11	*sll0356*	5'-phosphoribosyl anthranilate isomerase
	2178468-2178493	+	catttTtcag_GCGATcGcCGAT	A209	11	-	-
	2400367-2400392	+	tccgCccaTg_GCGATcGgCGAT	A208	10	*sll0771*	glucose transport protein
	2431716-2431741	-	gccttcaacT_GgGtTTGACGAT	A209	11	*slr0346*	ribonuclease III
	2446056-2446081	+	ATattgtcaT_GgGATTGACaAT	A207	9	-	-
	2541513-2541538	+	gTttagtcTg_GCGATcGcCGAT	A208	10	*sll0415*	ABC transporter
	2572081-2572106	-	tgccgcccTg_GCGATcGcCGAT	A209	11	*sll0068*	unknown protein
	2574029-2574054	-	taaggTGccg_GCGATcGcCGAT	A208	10	*sll0067*	glutathione S-transferase
	2588466-2588491	+	gcattgGtca_cCGATTGACGcT	A209	11	*sll0058*	DnaK protein
	2597048-2597073	-	ActtgTccca_cCGATTGgCGAT	A208	10	*slr0067*	ATP-binding protein involved in chromosome partitioning
	2942897-2942922	+	ggGgCattTg_GCGATcGcCGAT	A207	9	-	-
	2947562-2947587	+	ATtgCcctca_GCGATTtACGcT	A207	9	*slr0615*	ATP-binding cassette, subfamily B, multidrug efflux pump
	3212295-3212320	+	tccAaattgg_GCGATcGgCGAT	A209	11	*slr0541*	unknown protein
	3395838-3395863	+	ATGgaaaaTg_GCGATTaACcAT	A206	8	*sll1477*	unknown protein
	3412312-3412337	-	caacaatGga_cCGATTGgCGAT	A209	11	*sll0736*	unknown protein
	3520784-3520809	+	gTacCcaccT_GCGATcGcCGAT	A207	9	*sll1110*	peptide chain release factor
	3537459-3537484	-	ggtAtTGacg_GCGATcGcCGAT	A207	9	*sll0578*	phosphoribosyl aminoimidazole carboxylase
	3556832-3556857	-	gactggcaaa_GCcATTGgCGAT	A210	12	*sll0564*	unknown protein

sll0574	80660-80683	+	cattcCCA_GTTCAgCtTTGT	A205	7	*sll1056*	phosphoribosylformyl glycinamidine synthetase II
	114355-114378	-	GGcaAtt_GTTCACCaaTGT	A205	7	*slr0729*	unknown protein
	135400-135423	-	GGGccggc_GTTtgCCCTTGT	A205	7	*slr0744*	initiation factor IF-2
	219419-219442	-	GccGctCg_GgTCACCCTgGT	A205	7	*sll1029*	carbon dioxide concentrating mechanism protein
	322713-322736	-	ttGGAaCg_GTTtACCCaTGT	A204	6	*slr0963*	ferredoxin-sulfite reductase
	529560-529583	+	tGGagtgg_cTTCACCCTaGT	A206	8	*slr1753*	unknown protein
	602477-602500	+	ccGcttCg_GTTaACCtTTGT	A206	8	*sll1932*	DnaK protein
	803137-803160	-	ctGGAgtg_cTTCACCCTgGT	A205	7	*slr1143*	unknown protein
	835311-835334	+	GcGGAtgc_cTTtACCCTTGT	A204	6	*sll1810*	50S ribosomal protein L6
	1177960-1177983	+	aacaAtat_tTTCACCCTTGa	A207	9	*slr1403*	integrin alpha- and beta4- subunit domain homologue
	1564393-1564416	-	atccACCA_GTTCACCgaTGT	A204	6	*slr2098*	hybrid sensory kinase
	1613993-1614016	+	caccAggc_GTTtAtCCTTGT	A207	9	*slr1521*	GTP-binding protein
	2332627-2332650	+	ttccttat_GTTCACCtTTGc	A208	10	*sll0158*	1,4-alpha-glucan branching enzyme
	2359738-2359761	-	GaGtcCag_GTTCACCCgTGa	A205	7	-	-
	2547462-2547485	+	cGaGgagA_GTTaACCaTTGT	A205	7	*sll0409*	o-succinylbenzoate synthase
	2813544-2813567	-	cctccCtg_GTTaACCaTTGT	A207	9	*sll0545*	unknown protein
	2842647-2842670	-	tcccAttg_GTTCACCaTTGa	A207	9	-	-
	3006504-3006527	-	aGacgaCc_GTaCcCCCTTGT	A206	8	*sll0290*	polyphosphate kinase
	3007775-3007798	+	cactAaag_GTTCcCCtTTGT	A207	9	*sll0289*	septum site-determining protein
	3157718-3157741	+	GaattagA_GTTaACCCaTGT	A206	8	*slr0033*	aspartyl-tRNA(Asn)/glutamyl-tRNA(Gln) amidotransferase subunit C
	3247855-3247878	+	GGcactaA_GTTCACCCcTGg	A205	7	-	-
	3433031-3433054	-	cttGgCtA_GTTaACCCTTtT	A205	7	-	-
	3476773-3476796	-	GGaGAagc_GTctACCCTTGT	A204	6	*slr1668*	unknown protein
	3521819-3521842	+	atGtttgA_GTTCAtCaTTGT	A206	8	*sll1109*	unknown protein

Table S2. List of organisms and plasmids used/generated in this work.

Organism Name/Genotype.	Description	Source
Escherichia coli DH5α	Transformation/cloning strain	Invitrogen
Escherichia coli XL1-Blue	Transformation/cloning strain	Agilent
Synechocystis sp. PCC 6803	Wild-type substrain Kasuza	Pasteur Culture Collection
sll0574 mutant	*Synechocystis* mutant with *sll0574* (from 46 to 764 bp) replaced by a Km resistance cassette	This work
Syn dCas9	Δ*psbA1*::P_{psbA2} dCas9 SpR	This work
3-sgRNA *kpsM* mutant	Δ*psbA1*::P_{psbA2} dCas9 SpR; pLY::P_{L31} sgRNA-sll0574_15 slr0977_16 slr2107_56 CmR	This work
**Plasmid**	**Description**	**Source**
pGEM®-T easy	T/A cloning vector	Promega
pKm.1	pGEM-T easy with the Km resistance cassette	Pinto *et al*, 2015
pGDsll0574	pGEM-T easy habouring *sll0574*'s flanking sequences for double homologous recombination, including a XmaI site in between	This work
pGDsll0574.Km	pGDsll0574 with a Km resistance cassette inserted into the XmaI site	This work
pMD19T_psbA1_P_{psbA2}_dCas9_B0015_SpR	Plasmid to transform the dCas9 into *Synechocystis*	Yao *et al*, 2016
pMD19T_slr0230_slr0231_P_{L31}_sgRNA NT1_B0015_KmR	Assembly plasmid for the sgRNA arrays	Yao *et al*, 2016
pLY KmR	Replicative vector for *Synechocystis*	Kindly provided by Paul Hudson's group
pLY CmR	Replicative vector for *Synechocystis*	This work
pLY::P_{L31}_sgRNA-sll0574_15 slr0977_16 slr2107_56 CmR	Replicative vector for *Synechocystis* transformation	This work

Table S3. Primer nucleotide sequences and annealing temperatures (T_a) used in RT-qPCR.

Gene	Primer Name	Primer Sequence (5′- 3′)	T_a (°C)	Source
sll0574	sll0574_RTq_Fwd	CCGGCACAATTTCGGATGG	56	This work
	sll0574_RTq_Rev	CCCTCTCCATGATCGTCGC		
slr2107	slr2107F(Ra)	GACCCATCGTCAATCGCAAC	56	This work
	slr2107RT	CACATCCTTCGCCACCAA		
slr0977	slr0977F	CGCACGGAGCGTCAGTATT	56	This work
	slr0977RO	CCGCAAACACCAGAATGGGAT		
rrn16Sa.b	BD16SF1	CACACTGGGACTGAGACAC	56	Pinto *et al*, 2012
	BD16SR1	CTGCTGGCACGGAGTTAG		
petB	SpetB1F	CCTTCGCCTCTGTCCAATAC	56	
	SpetB1R	TAGCATTACACCCACAACCC		
rnpB	rnpBF1	CGTTAGGATAGTGCCACAG	56	
	rnpBR1	CGCTCTTACCGCACCTTTG		

Table S4. Primer nucleotide sequences used to verify the segregation of the *Syn* dCas9 strain.

Primer Name	Primer Sequence (5′-3′)	Source
dCas9i_Fwd	GTTTTGCCAATCGCAATTTT	This work
dCas9i_Rev	CACGTGCCATTTCAATAACG	
psbA1_Rev	AACCAAGGAACCGTGCATAG	
FR_Fwd	GCCACAACCAGGCAGTATTT	
FR_Rev	CCAGGCAATCCACTGATTTT	
